# Fifty Years of Eating Disorder Research: Growth, Gaps, and Global Challenges

**DOI:** 10.1002/eat.24499

**Published:** 2025-07-02

**Authors:** Anja Hilbert

**Affiliations:** ^1^ Behavioral Medicine Research Unit, Integrated Research and Treatment Center AdiposityDiseases, Department of Psychosomatic Medicine and Psychotherapy University of Leipzig Medical Center Leipzig Germany

**Keywords:** altmetric attention score, bibliometric analysis, citation analysis, eating disorder, feeding and eating disorders, public attention

## Abstract

The bibliometric study by Lee and Chi presents the most comprehensive analysis of eating disorder (ED) research publications to date, tracing publication trends, thematic developments, and the interplay between academic and public attention over the past five decades. The findings reveal a marked and sustained growth in ED‐related publications since the early 2000s, reflecting the field's consolidation while also underscoring persistent imbalances. Notably, the analysis highlights the dominance of English‐speaking countries in research output, the underrepresentation of lower‐income regions, and the limited visibility of certain EDs and demographic groups. Network analyses of Medical Subject Headings' terms indicate thematic stability over time but also point to neglected areas of clinical relevance. Furthermore, the weak correlation between academic citations and public attention raises important questions about science communication and the alignment of scholarly impact with societal salience. To enhance global inclusivity and scientific relevance, it is proposed that publication barriers be removed, thematic scope broadened, and cross‐disciplinary collaboration strengthened. Such strategies are essential to enable ED research to remain responsive to evolving clinical needs within culturally and demographically diverse populations amid an increasingly complex global landscape.

The bibliometric analysis by Lee and Chi (2025) provides a comprehensive mapping of trends in eating disorder (ED) research spanning five decades, the longest period studied to date. The study centered on publication trends, core research themes and developments, and the interaction between public and academic attention. One of the most striking findings is the marked and sustained growth in ED‐related publications since the early 2000s. This growth underscores the establishment of the research field and may be interpreted as a sign of progress in recognition and care. However, it also raises critical questions about the geographical inclusivity, thematic scope, and publishing culture in ED research.

## 1 The Development of the ED Research Field

Lee and Chi (2025) identify the *International Journal of Eating Disorders* as a cornerstone in the development of ED research, consistently publishing the highest volume of ED‐specific publications since its inception in 1981. Founded in the United States, the journal reflects the dominance of US‐based research—the largest contributor to global scientific output—followed by contributions from other English‐speaking countries such as the United Kingdom, Australia, and Canada, where language‐related barriers to publication are minimal. Encouragingly, research contributions from non‐English‐speaking countries have been increasing, with growing representation from other European countries, Turkey, and China. The rise of geographically diverse journals, including the European journals *European Eating Disorders Review* and *Eating and Weight Disorders* since the 1990s, and Australia's *Journal of Eating Disorders* since 2013—the field's first fully open‐access journal—has further supported international proliferation. Journals with broader scopes (e.g., *Appetite*, *Eating Behaviors*, *Body Image*) across high‐income regions are also publishing more ED‐related work. This diversification reflects a stabilizing research landscape amid global polycrises and signals a shift toward a more globalized perspective—an important development given the cultural variability of ED symptomatology.

A prior bibliometric analysis spanning four decades of ED research documented increased output after 2000 in both North America and, more notably, Europe, but only limited growth in other regions, especially low‐income countries (He et al. 2022). Persistent challenges—such as language barriers and limited research funding—may hinder progress. To counteract disparities and reduce publication bias, targeted strategies were devised. The *International Journal of Eating Disorders*, for example, has issued special calls for submissions from underrepresented regions (e.g., Latin America, Asia) and diversified its editorial leadership to foster regional engagement. This diversification involves connecting researchers—including early‐career scholars—with local editors for guidance on manuscript submission and opportunities to participate in the editorial process (e.g., through ad hoc reviews or editorial board membership). Further, resources on scientific writing and reviewing (e.g., workshops, webinars) and language editing tools have been provided. Open‐access discounts have also been negotiated for many countries. While the impact of these efforts has not been investigated, increasing contributions from non‐English‐speaking countries (Lee and Chi 2025) point to some progress. Future efforts should continue editorial diversification by appointing regional representatives and offering open‐access publication discounts to researchers from additional countries. Another promising approach involves publishing open‐access practical reviews on emerging methodologies (e.g., computational modeling) to provide timely and globally accessible research guidance.

Importantly, promoting global research equity is not the responsibility of journals alone. Funding bodies and scientific societies must also foster international collaboration by supporting cross‐national research initiatives, exchange programs, and inclusive scientific meetings. Ensuring that conferences are accessible to researchers in lower‐income countries—including by hosting events locally or offering virtual and hybrid formats—can help build a more globally interconnected ED research community. Looking ahead, artificial intelligence (AI) could increasingly support reducing barriers in conducting and publishing research. However, this potential hinges on the responsible use of high‐quality, transparent, and ethically developed AI tools (Linardon and Fuller‐Tyszkiewicz 2025).

Beyond the growing global diversification of ED research, it is important to note that EDs remain underrepresented in multidisciplinary or general psychiatric, psychological, and medical journals. Despite advances in establishing ED research as a distinct field, funding analyses from countries such as the United States, Canada, and Australia indicate that ED research receives disproportionately less funding relative to other disorders of comparable prevalence and severity (Haynos et al. 2024). This disparity may partly reflect enduring gendered stigma, with EDs often viewed as a “women's issue,” which may result in marginalization compared to more widely acknowledged disorders like schizophrenia. Such stigmatization appears to influence academic publishing: ED research often remains “siloed” and overlooked by adjacent disciplines, adversely affecting not only academic careers but also the broader understanding, diagnosis, and treatment of EDs. Investigating how researchers in related fields perceive the relevance of EDs could uncover potential misconceptions and stereotype‐driven biases. Moreover, a systematic analysis of ED research visibility in nonspecialized journals could help identify barriers and inform strategies to enhance integration into the wider scientific community.

Although the growing volume of ED‐related publications is a positive trend, concerns about research quality and integrity have emerged. Lee and Chi (2025) document the rise of potential “predatory journals”—open‐access venues that charge high publication fees while offering minimal editorial oversight and peer review (Laine et al. 2025)—in the ED field. Such platforms may enable the dissemination of low‐quality or even fraudulent studies, including those produced by “paper mills” that generate manuscripts for profit. These trends reflect broader systemic pressures in academia, including the “publish or perish” culture and an overreliance on journal‐based metrics like the journal impact factor in evaluations and funding decisions. Metrics such as the journal impact factor are influenced by field size, which places ED research at a disadvantage compared to larger fields. Alternative, more nuanced metrics—such as the field‐normalized journal citation indicator or article‐level impact measures—may offer a more appropriate basis for evaluation.

## 2 Thematic Stability and Persistent Gaps

Through a network analysis of Medical Subject Headings (MeSH), Lee and Chi (2025) offer a retrospective “constellational” mapping of the ED scientific landscape, highlighting both thematically stable and underexplored domains—despite their clinical relevance. Core MeSH terms such as “humans,” “female,” “adolescent,” and “adult” have remained dominant across five decades. Although broader categories like *feeding and eating disorders* have gained visibility, a relative decline in publications on anorexia nervosa was observed, historically regarded as the paradigmatic ED, suggesting a thematic broadening of ED research.

In contrast, other EDs are only sporadically represented in the network visualizations of key MeSH terms, despite their prevalence and clinical importance. Notably absent are conditions such as *night eating syndrome*, *purging disorder*, *rumination disorder*, and *avoidant/restrictive food intake disorder*. This gap is likely due to the more recent formal recognition of these disorders and—for some—their lower prevalence compared to “traditional” EDs. However, a mismatch between research attention and clinical needs cannot be ruled out. Given the bump chart's limitation to the 10 most frequent MeSH terms, it remains unclear whether these underrepresented disorders have gained visibility over time. Future bibliometric analyses should examine lower‐frequency MeSH terms to more accurately assess the field's responsiveness to shifting clinical priorities.

A further critical gap lies in the demographic scope of ED. The predominance of research focused on females and adolescents reflects not only the clinical distribution of EDs but also entrenched biases in research priorities. The underrepresentation of males, children, and middle‐aged adults limits the generalizability of findings and may perpetuate diagnostic and treatment disparities. Broadening demographic representation must therefore be a priority in future research agendas, particularly in light of growing awareness that EDs affect individuals across all genders and age groups.

To address these gaps, the *International Journal of Eating Disorders*, for example, has implemented Demographic Reporting Guidelines requiring authors to justify any omission of key demographic variables and to discuss the impact on generalizability. Further, all ED journals have issued special calls on several neglected topics. Future initiatives could emphasize calls or review series dedicated to underrepresented conditions—such as night eating syndrome, loss of control eating, or early‐onset feeding disorders beyond avoidant/restrictive food intake disorder. This may be especially timely in light of forthcoming revisions to the *Diagnostic and Statistical Manual of Mental Disorders*. Additionally, emerging areas such as feeding or EDs associated with undernutrition influenced by external conditions (e.g., poverty, displacement, or environmental crises) warrant increased scholarly attention to reflect the evolving nature and global diversity of ED presentations.

Overall, the potential disconnect between research focus and clinical need highlights the importance of integrating insights from frontline clinical care and patient experience. Clinicians often identify emerging issues or challenge existing categories, while patients provide valuable perspectives on unmet needs and complex lived experiences. Incorporating these voices—through qualitative methods, clinical observation, and participatory approaches—can help align research with real‐world complexities and ensure its clinical relevance.

## 3 Academic and Public Attention: Divergent Trajectories

Analysis of citation and Altmetric trends reveals dynamic yet divergent patterns. While both metrics show an encouraging upward trajectory, their negligible correlation indicates only minimal overlap between academic and public attention. This disconnect between scholarly influence and societal resonance highlights challenges in science communication, often reflecting topical urgency or sensationalism rather than scientific merit. As Lee and Chi (2025) emphasize, although public engagement is vital for awareness, advocacy, and funding, it should not be conflated with scientific quality. To address the disconnect between scientific quality and public attention, science communication must make rigorous research accessible without resorting to simplification or sensationalism. Potential strategies include expert‐led explainers of relevant research (e.g., podcasts), lay summaries in publications as realized by most ED journals, and open public dialogue, for which researchers could be trained and incentivized. Enhancing media literacy, for example, regarding the interpretation of metrics such as altmetrics, is also essential to prevent misrepresentation of a study's impact.

Of potential concern is the observed decline in mean citations per year since 2019. While citations accrue over time, this trend may reflect structural pressures within academia. Favoring publication volume over impact may contribute to a saturated literature landscape with limited scholarly uptake. Further, short‐form publication formats—such as scoping reviews, research letters, and commentaries—serve valid and, at times, strategically important functions in scholarly communication. Nonetheless, if not balanced by rigorous and impactful research, their proliferation risks diluting citation metrics and fragmenting citation behaviors, potentially contributing to a broader but shallower academic discourse, a phenomenon that merits critical reflection in journal editorial policies.

## 4 Conclusions and Future Directions

Five decades of ED research show the field's impressive growth but geographical, thematic, and formal imbalances. To foster global representation, journals publishing ED research should continue to internationalize their editorial boards and author bases by actively engaging researchers from underrepresented regions and supporting initiatives to overcome language barriers. Expanding the thematic scope to encompass the full spectrum of EDs and clinically relevant eating disturbances—while integrating perspectives from both clinicians and patients—requires support from both journals and funding agencies. Particular attention must be given to underrepresented demographic groups to ensure that research reflects the full heterogeneity of ED presentations. A comprehensive understanding necessitates the investigation of a broad spectrum of sociodemographic and psychosocial factors—such as sexual orientation, ethnicity, migration status, socioeconomic variables, and environmental pressures resulting from the current polycrises (e.g., displacement, hunger, trauma). Furthermore, promoting cross‐disciplinary collaboration is essential for enhancing the visibility and recognition of ED research. This could be advanced through ED special issues in general psychiatric journals, inclusive of diverse disciplinary perspectives, and through funding schemes that explicitly support interdisciplinary partnerships. In an era characterized by information overload, misinformation, and complex global challenges, high‐quality science communication is vital for fostering informed public discourse, strengthening societal trust in research, and supporting evidence‐based policymaking.

Building on its achievements over the past five decades, the ED research community is well positioned to strengthen its vibrant and impactful field—one that is responsive to evolving scientific gaps and the pressing clinical needs of diverse populations worldwide.

## Author Contributions

**Anja Hilbert:** conceptualization, writing – original draft, writing – review and editing.

## Conflicts of Interest

Anja Hilbert reports receiving research grants from the German Federal Ministry of Education and Research, German Research Foundation, Innovation Fund, and Roland Ernst Foundation for Health Care; royalties for books on the treatment of eating disorders and obesity with Hogrefe and Kohlhammer; honoraria for workshops and lectures on eating disorders and obesity and their treatment, including Lilly and Novo Nordisk; honoraria as editor of the *International Journal of Eating Disorders* and the journal *Psychotherapeut*; honoraria as a reviewer from Oxford University Press and the German Society for Nutrition; and honoraria as a consultant for WeightWatchers, Zweites Deutsches Fernsehen, and Takeda.

## Data Availability

Data sharing is not applicable to this article as no new data were created or analyzed in this study.
